# Supplementary material for: EnzML: multi-label prediction of enzyme classes using InterPro signatures
Source: BMC Bioinformatics. 2012 Apr 25;13:61. doi: 10.1186/1471-2105-13-61 (PMC3483700; doi:10.1186/1471-2105-13-61)
Supplement: Addtional file 5 — The Java code to format the data files, evaluate and predict. The file enzml_java_code.tar.gz contains the Java code used to format database data to ARFF and XML formats, to execute cross and train-test (jackknife) evaluations and to record evaluation results to database. More information is included in the readme.txt file and the Javadoc files. The code can be used with a MySQL database. To use a different database software, other JDBC drivers might be required. [file 1471-2105-13-61-S5.gz › java_code/enzml2011/doc/uk/ac/ed/inf/csb/taweka2010/package-summary.html]

uk.ac.ed.inf.csb.taweka2010


---


|  |  |  |
| --- | --- | --- |
| **Package** \| Class \| **Use** \| **Tree** \| **Deprecated** \| **Index** \| **Help** | |  |
| PREV PACKAGE   NEXT PACKAGE | **FRAMES**    **NO FRAMES**     **All Classes** |  |


---

## Package uk.ac.ed.inf.csb.taweka2010

| **Class Summary** | |
| --- | --- |
| **AllTests** | Class |
| **AttributeFactory** | Attribute factory. |
| **AttributeFactoryTest** | Class |
| **DataSetFactory** | DataSet contains a collection of instances (rows), their attributes (column names) and their attribute values (cells) The last attribute is the class attribute. |
| **DataSetFactoryTest** | Class |
| **FastVectorUtils** | Utilities for converting between normal vectors/enumerators to Weka FastVector |
| **InstancesFactory** | Generates and fill instances for a weka data set |
| **InstancesFactoryTest** | Class |
| **TaWeka** | Class |
| **WekaTest** | Class to test basic weka instance creation |

---


|  |  |  |
| --- | --- | --- |
| **Package** \| Class \| **Use** \| **Tree** \| **Deprecated** \| **Index** \| **Help** | |  |
| PREV PACKAGE   NEXT PACKAGE | **FRAMES**    **NO FRAMES**     **All Classes** |  |


---
